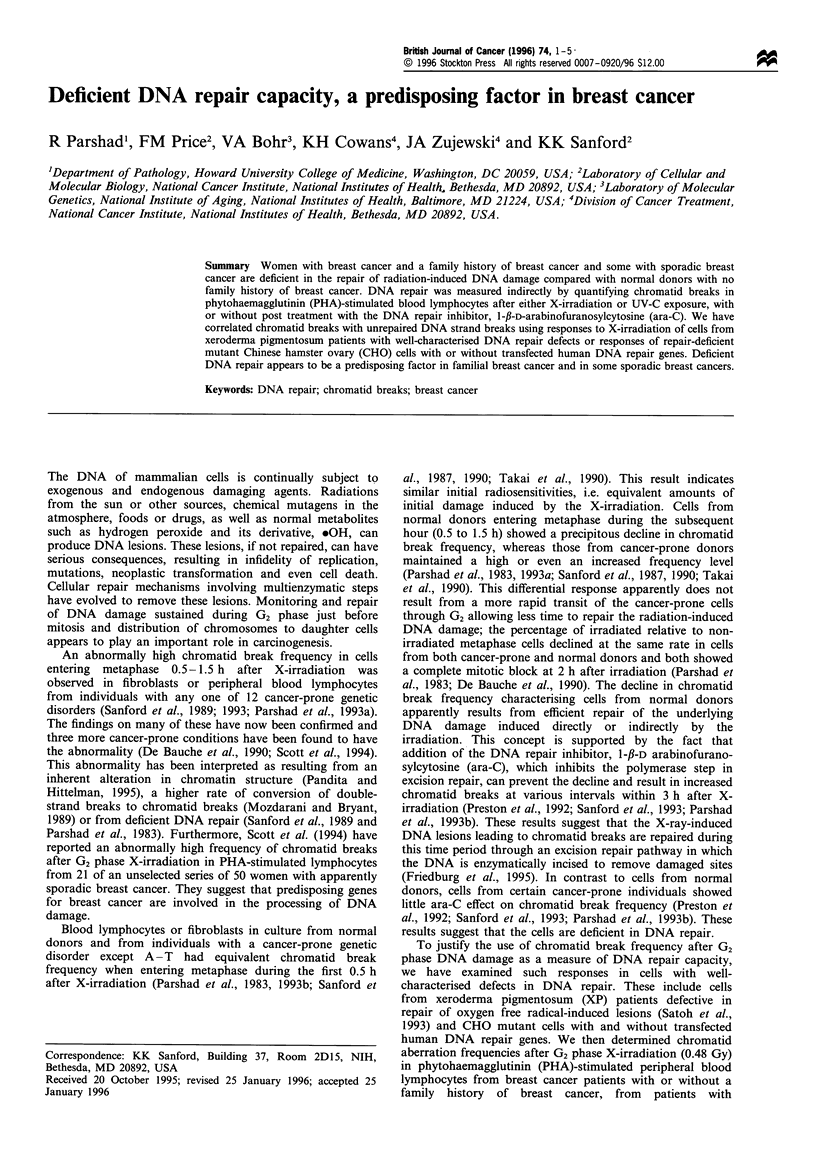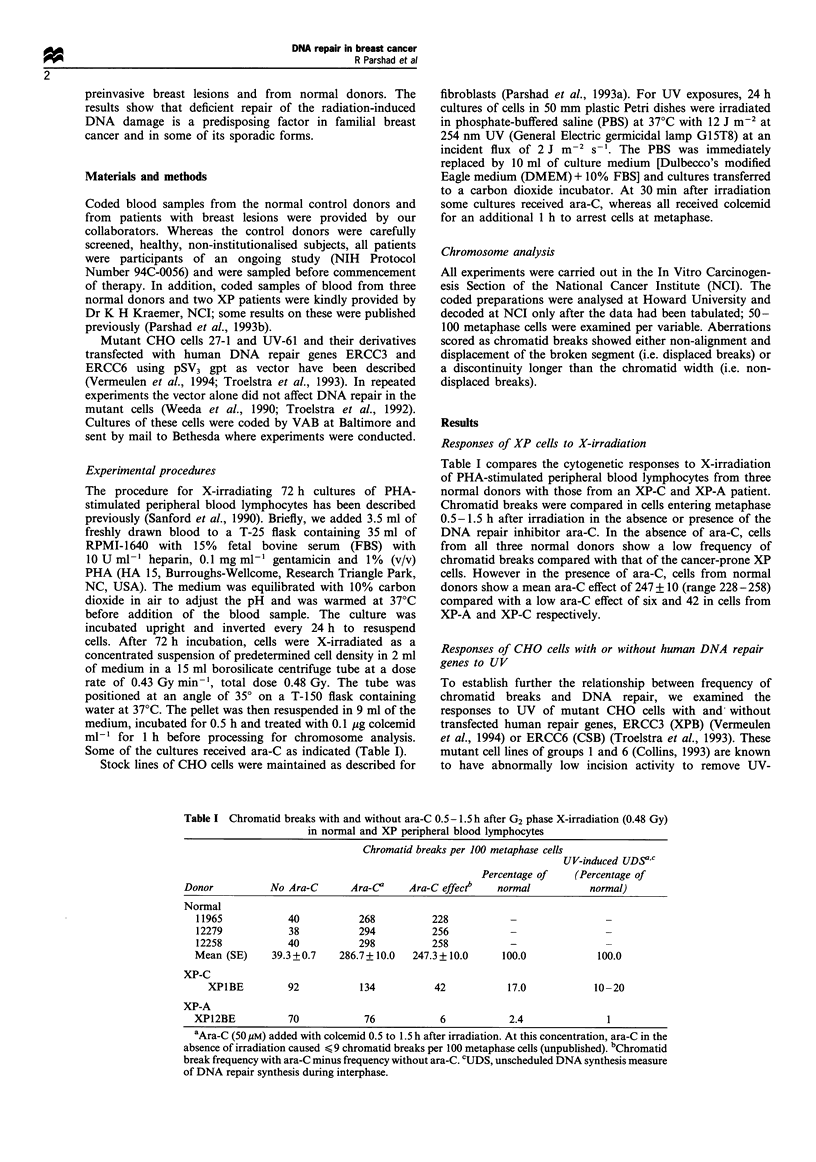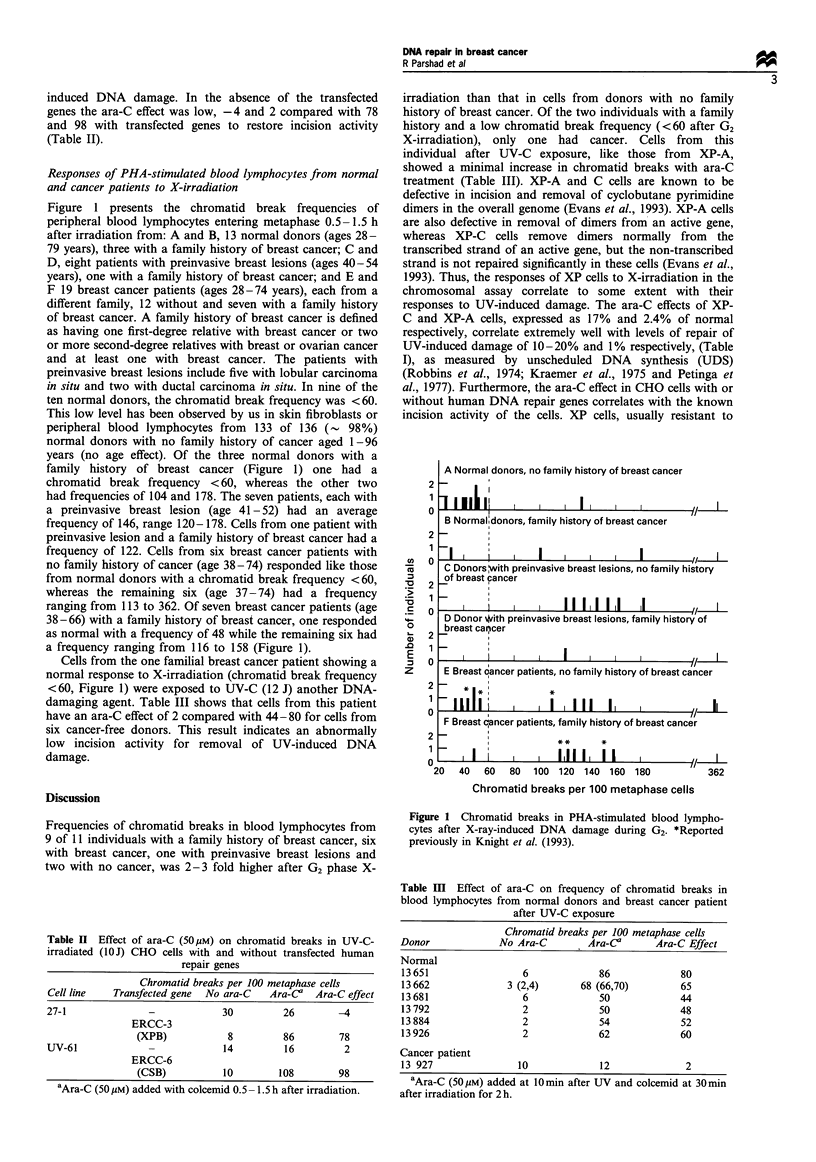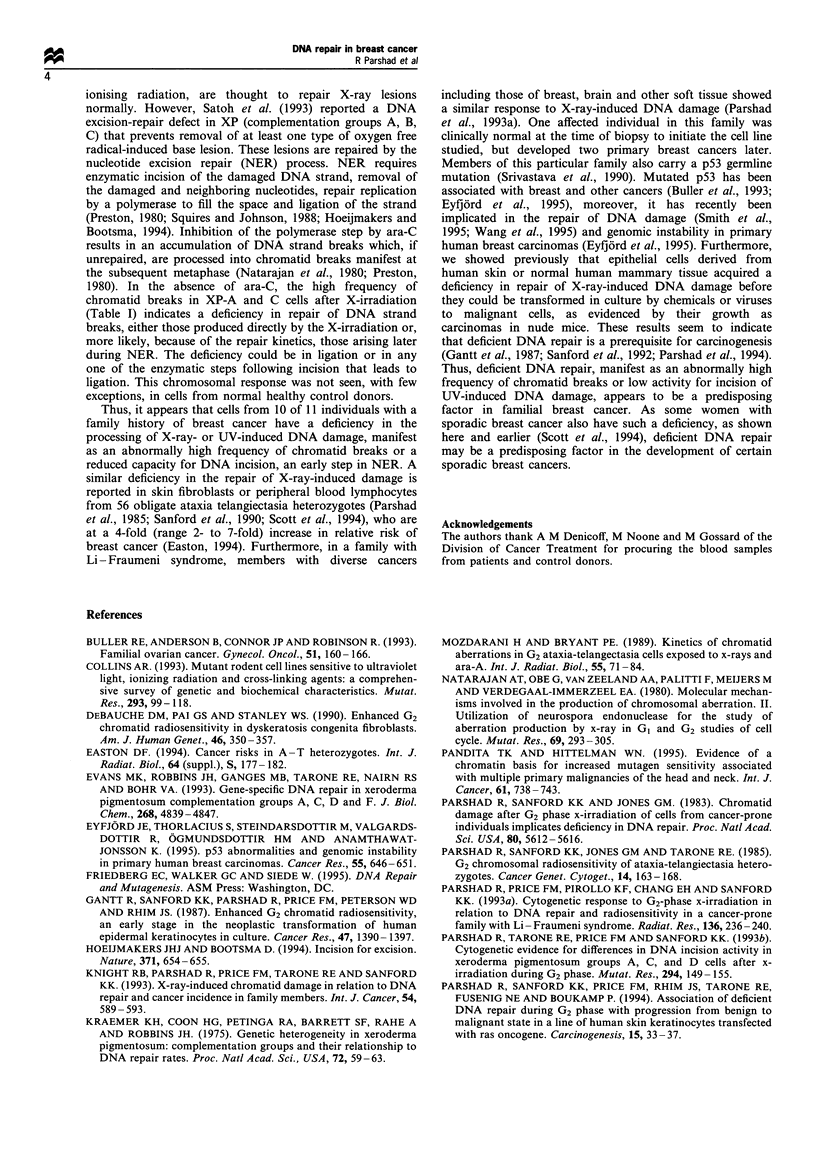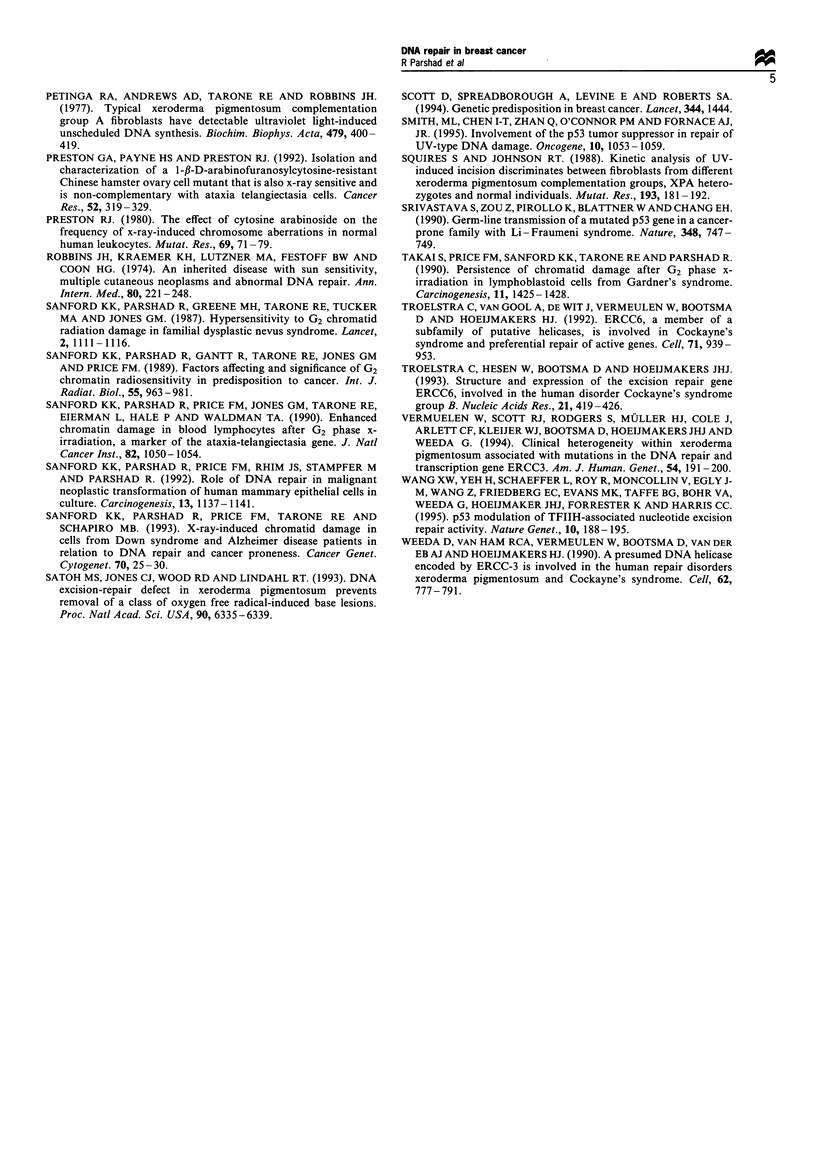# Deficient DNA repair capacity, a predisposing factor in breast cancer.

**DOI:** 10.1038/bjc.1996.307

**Published:** 1996-07

**Authors:** R. Parshad, F. M. Price, V. A. Bohr, K. H. Cowans, J. A. Zujewski, K. K. Sanford

**Affiliations:** Department of Pathology, Howard University College of Medicine, Washington, DC 20059, USA.

## Abstract

Women with breast cancer and a family history of breast cancer and some with sporadic breast cancer are deficient in the repair of radiation-induced DNA damage compared with normal donors with no family history of breast cancer. DNA repair was measured indirectly by quantifying chromatid breaks in phytohaemagglutinin (PHA)-stimulated blood lymphocytes after either X-irradiation or UV-C exposure, with or without post treatment with the DNA repair inhibitor, 1-beta-D-arabinofuranosylcytosine (ara-C). We have correlated chromatid breaks with unrepaired DNA strand breaks using responses to X-irradiation of cells from xeroderma pigmentosum patients with well-characterised DNA repair defects or responses of repair-deficient mutant Chinese hamster ovary (CHO) cells with or without transfected human DNA repair genes. Deficient DNA repair appears to be a predisposing factor in familial breast cancer and in some sporadic breast cancers.


British Journal of Cancer (1996) 74, 1-5
© 1996 Stockton Press All rights reserved 0007-0920/96 $12.00

### Deficient DNA repair capacity, a predisposing factor in breast cancer

R Parshad[1], FM Price[2], VA Bohr[3], KH Cowans[4], JA Zujewski[4] and KK Sanford[2]

[1]*Department of Pathology, Howard University College of Medicine, Washington, DC 20059, USA;* [2]*Laboratory of Cellular and Molecular Biology, National Cancer Institute, National Institutes of Health, Bethesda, MD 20892, USA;* [3]*Laboratory of Molecular Genetics, National Institute of Aging, National Institutes of Health, Baltimore, MD 21224, USA;* [4]*Division of Cancer Treatment, National Cancer Institute, National Institutes of Health, Bethesda, MD 20892, USA.*

**Summary** Women with breast cancer and a family history of breast cancer and some with sporadic breast cancer are deficient in the repair of radiation-induced DNA damage compared with normal donors with no family history of breast cancer. DNA repair was measured indirectly by quantifying chromatid breaks in phytohaemagglutinin (PHA)-stimulated blood lymphocytes after either X-irradiation or UV-C exposure, with or without post treatment with the DNA repair inhibitor, 1-$\beta$-D-arabinofuranosylcytosine (ara-C). We have correlated chromatid breaks with unrepaired DNA strand breaks using responses to X-irradiation of cells from xeroderma pigmentosum patients with well-characterised DNA repair defects or responses of repair-deficient mutant Chinese hamster ovary (CHO) cells with or without transfected human DNA repair genes. Deficient DNA repair appears to be a predisposing factor in familial breast cancer and in some sporadic breast cancers.

**Keywords:** DNA repair; chromatid breaks; breast cancer

The DNA of mammalian cells is continually subject to exogenous and endogenous damaging agents. Radiations from the sun or other sources, chemical mutagens in the atmosphere, foods or drugs, as well as normal metabolites such as hydrogen peroxide and its derivative, •OH, can produce DNA lesions. These lesions, if not repaired, can have serious consequences, resulting in infidelity of replication, mutations, neoplastic transformation and even cell death. Cellular repair mechanisms involving multienzymatic steps have evolved to remove these lesions. Monitoring and repair of DNA damage sustained during $G_2$ phase just before mitosis and distribution of chromosomes to daughter cells appears to play an important role in carcinogenesis.

An abnormally high chromatid break frequency in cells entering metaphase 0.5–1.5 h after X-irradiation was observed in fibroblasts or peripheral blood lymphocytes from individuals with any one of 12 cancer-prone genetic disorders (Sanford *et al.*, 1989; 1993; Parshad *et al.*, 1993a). The findings on many of these have now been confirmed and three more cancer-prone conditions have been found to have the abnormality (De Bauche *et al.*, 1990; Scott *et al.*, 1994). This abnormality has been interpreted as resulting from an inherent alteration in chromatin structure (Pandita and Hittelman, 1995), a higher rate of conversion of double-strand breaks to chromatid breaks (Mozdarani and Bryant, 1989) or from deficient DNA repair (Sanford *et al.*, 1989 and Parshad *et al.*, 1983). Furthermore, Scott *et al.* (1994) have reported an abnormally high frequency of chromatid breaks after $G_2$ phase X-irradiation in PHA-stimulated lymphocytes from 21 of an unselected series of 50 women with apparently sporadic breast cancer. They suggest that predisposing genes for breast cancer are involved in the processing of DNA damage.

Blood lymphocytes or fibroblasts in culture from normal donors and from individuals with a cancer-prone genetic disorder except A–T had equivalent chromatid break frequency when entering metaphase during the first 0.5 h after X-irradiation (Parshad *et al.*, 1983, 1993b; Sanford *et al.*, 1987, 1990; Takai *et al.*, 1990). This result indicates similar initial radiosensitivities, i.e. equivalent amounts of initial damage induced by the X-irradiation. Cells from normal donors entering metaphase during the subsequent hour (0.5 to 1.5 h) showed a precipitous decline in chromatid break frequency, whereas those from cancer-prone donors maintained a high or even an increased frequency level (Parshad *et al.*, 1983, 1993*a*; Sanford *et al.*, 1987, 1990; Takai *et al.*, 1990). This differential response apparently does not result from a more rapid transit of the cancer-prone cells through $G_2$ allowing less time to repair the radiation-induced DNA damage; the percentage of irradiated relative to non-irradiated metaphase cells declined at the same rate in cells from both cancer-prone and normal donors and both showed a complete mitotic block at 2 h after irradiation (Parshad *et al.*, 1983; De Bauche *et al.*, 1990). The decline in chromatid break frequency characterising cells from normal donors apparently results from efficient repair of the underlying DNA damage induced directly or indirectly by the irradiation. This concept is supported by the fact that addition of the DNA repair inhibitor, 1-$\beta$-D arabinofuranosylcytosine (ara-C), which inhibits the polymerase step in excision repair, can prevent the decline and result in increased chromatid breaks at various intervals within 3 h after X-irradiation (Preston *et al.*, 1992; Sanford *et al.*, 1993; Parshad *et al.*, 1993b). These results suggest that the X-ray-induced DNA lesions leading to chromatid breaks are repaired during this time period through an excision repair pathway in which the DNA is enzymatically incised to remove damaged sites (Friedburg *et al.*, 1995). In contrast to cells from normal donors, cells from certain cancer-prone individuals showed little ara-C effect on chromatid break frequency (Preston *et al.*, 1992; Sanford *et al.*, 1993; Parshad *et al.*, 1993b). These results suggest that the cells are deficient in DNA repair.

To justify the use of chromatid break frequency after $G_2$ phase DNA damage as a measure of DNA repair capacity, we have examined such responses in cells with well-characterised defects in DNA repair. These include cells from xeroderma pigmentosum (XP) patients defective in repair of oxygen free radical-induced lesions (Satoh *et al.*, 1993) and CHO mutant cells with and without transfected human DNA repair genes. We then determined chromatid aberration frequencies after $G_2$ phase X-irradiation (0.48 Gy) in phytohaemagglutinin (PHA)-stimulated peripheral blood lymphocytes from breast cancer patients with or without a family history of breast cancer, from patients with

Correspondence: KK Sanford, Building 37, Room 2D15, NIH, Bethesda, MD 20892, USA
Received 20 October 1995; revised 25 January 1996; accepted 25 January 1996

preinvasive breast lesions and from normal donors. The results show that deficient repair of the radiation-induced DNA damage is a predisposing factor in familial breast cancer and in some of its sporadic forms.

### Materials and methods

Coded blood samples from the normal control donors and from patients with breast lesions were provided by our collaborators. Whereas the control donors were carefully screened, healthy, non-institutionalised subjects, all patients were participants of an ongoing study (NIH Protocol Number 94C-0056) and were sampled before commencement of therapy. In addition, coded samples of blood from three normal donors and two XP patients were kindly provided by Dr K H Kraemer, NCI; some results on these were published previously (Parshad *et al.*, 1993b).

Mutant CHO cells 27-1 and UV-61 and their derivatives transfected with human DNA repair genes ERCC3 and ERCC6 using $pSV_3$ gpt as vector have been described (Vermeulen *et al.*, 1994; Troelstra *et al.*, 1993). In repeated experiments the vector alone did not affect DNA repair in the mutant cells (Weeda *et al.*, 1990; Troelstra *et al.*, 1992). Cultures of these cells were coded by VAB at Baltimore and sent by mail to Bethesda where experiments were conducted.

### *Experimental procedures*

The procedure for X-irradiating 72 h cultures of PHA-stimulated peripheral blood lymphocytes has been described previously (Sanford *et al.*, 1990). Briefly, we added 3.5 ml of freshly drawn blood to a T-25 flask containing 35 ml of RPMI-1640 with 15% fetal bovine serum (FBS) with 10 U $ml^{-1}$ heparin, 0.1 mg $ml^{-1}$ gentamicin and 1% (v/v) PHA (HA 15, Burroughs-Wellcome, Research Triangle Park, NC, USA). The medium was equilibrated with 10% carbon dioxide in air to adjust the pH and was warmed at 37°C before addition of the blood sample. The culture was incubated upright and inverted every 24 h to resuspend cells. After 72 h incubation, cells were X-irradiated as a concentrated suspension of predetermined cell density in 2 ml of medium in a 15 ml borosilicate centrifuge tube at a dose rate of 0.43 Gy $min^{-1}$, total dose 0.48 Gy. The tube was positioned at an angle of 35° on a T-150 flask containing water at 37°C. The pellet was then resuspended in 9 ml of the medium, incubated for 0.5 h and treated with 0.1 $\mu$g colcemid $ml^{-1}$ for 1 h before processing for chromosome analysis. Some of the cultures received ara-C as indicated (Table I).

Stock lines of CHO cells were maintained as described for fibroblasts (Parshad *et al.*, 1993a). For UV exposures, 24 h cultures of cells in 50 mm plastic Petri dishes were irradiated in phosphate-buffered saline (PBS) at 37°C with 12 J $m^{-2}$ at 254 nm UV (General Electric germicidal lamp G15T8) at an incident flux of 2 J $m^{-2}$ $s^{-1}$. The PBS was immediately replaced by 10 ml of culture medium [Dulbecco's modified Eagle medium (DMEM) + 10% FBS] and cultures transferred to a carbon dioxide incubator. At 30 min after irradiation some cultures received ara-C, whereas all received colcemid for an additional 1 h to arrest cells at metaphase.

### *Chromosome analysis*

All experiments were carried out in the In Vitro Carcinogenesis Section of the National Cancer Institute (NCI). The coded preparations were analysed at Howard University and decoded at NCI only after the data had been tabulated; 50–100 metaphase cells were examined per variable. Aberrations scored as chromatid breaks showed either non-alignment and displacement of the broken segment (i.e. displaced breaks) or a discontinuity longer than the chromatid width (i.e. non-displaced breaks).

### Results

### *Responses of XP cells to X-irradiation*

Table I compares the cytogenetic responses to X-irradiation of PHA-stimulated peripheral blood lymphocytes from three normal donors with those from an XP-C and XP-A patient. Chromatid breaks were compared in cells entering metaphase 0.5–1.5 h after irradiation in the absence or presence of the DNA repair inhibitor ara-C. In the absence of ara-C, cells from all three normal donors show a low frequency of chromatid breaks compared with that of the cancer-prone XP cells. However in the presence of ara-C, cells from normal donors show a mean ara-C effect of 247 ± 10 (range 228–258) compared with a low ara-C effect of six and 42 in cells from XP-A and XP-C respectively.

### *Responses of CHO cells with or without human DNA repair genes to UV*

To establish further the relationship between frequency of chromatid breaks and DNA repair, we examined the responses to UV of mutant CHO cells with and without transfected human repair genes, ERCC3 (XPB) (Vermeulen *et al.*, 1994) or ERCC6 (CSB) (Troelstra *et al.*, 1993). These mutant cell lines of groups 1 and 6 (Collins, 1993) are known to have abnormally low incision activity to remove UV-

**Table I** Chromatid breaks with and without ara-C 0.5–1.5 h after $G_2$ phase X-irradiation (0.48 Gy) in normal and XP peripheral blood lymphocytes

| | *Chromatid breaks per 100 metaphase cells* | | | | |
|---|---|---|---|---|---|
| *Donor* | *No Ara-C* | *Ara-C*[a] | *Ara-C effect*[b] | *Percentage of normal* | *UV-induced UDS*[a,c] *(Percentage of normal)* |
| Normal | | | | | |
| 11965 | 40 | 268 | 228 | – | – |
| 12279 | 38 | 294 | 256 | – | – |
| 12258 | 40 | 298 | 258 | – | – |
| Mean (SE) | 39.3 ± 0.7 | 286.7 ± 10.0 | 247.3 ± 10.0 | 100.0 | 100.0 |
| XP-C | | | | | |
| XP1BE | 92 | 134 | 42 | 17.0 | 10–20 |
| XP-A | | | | | |
| XP12BE | 70 | 76 | 6 | 2.4 | 1 |

[a]Ara-C (50 $\mu$M) added with colcemid 0.5 to 1.5 h after irradiation. At this concentration, ara-C in the absence of irradiation caused ⩽9 chromatid breaks per 100 metaphase cells (unpublished). [b]Chromatid break frequency with ara-C minus frequency without ara-C. [c]UDS, unscheduled DNA synthesis measure of DNA repair synthesis during interphase.

induced DNA damage. In the absence of the transfected genes the ara-C effect was low, −4 and 2 compared with 78 and 98 with transfected genes to restore incision activity (Table II).

*Responses of PHA-stimulated blood lymphocytes from normal and cancer patients to X-irradiation*

Figure 1 presents the chromatid break frequencies of peripheral blood lymphocytes entering metaphase 0.5–1.5 h after irradiation from: A and B, 13 normal donors (ages 28–79 years), three with a family history of breast cancer; C and D, eight patients with preinvasive breast lesions (ages 40–54 years), one with a family history of breast cancer; and E and F 19 breast cancer patients (ages 28–74 years), each from a different family, 12 without and seven with a family history of breast cancer. A family history of breast cancer is defined as having one first-degree relative with breast cancer or two or more second-degree relatives with breast or ovarian cancer and at least one with breast cancer. The patients with preinvasive breast lesions include five with lobular carcinoma *in situ* and two with ductal carcinoma *in situ*. In nine of the ten normal donors, the chromatid break frequency was <60. This low level has been observed by us in skin fibroblasts or peripheral blood lymphocytes from 133 of 136 (∼ 98%) normal donors with no family history of cancer aged 1–96 years (no age effect). Of the three normal donors with a family history of breast cancer (Figure 1) one had a chromatid break frequency <60, whereas the other two had frequencies of 104 and 178. The seven patients, each with a preinvasive breast lesion (age 41–52) had an average frequency of 146, range 120–178. Cells from one patient with preinvasive lesion and a family history of breast cancer had a frequency of 122. Cells from six breast cancer patients with no family history of cancer (age 38–74) responded like those from normal donors with a chromatid break frequency <60, whereas the remaining six (age 37–74) had a frequency ranging from 113 to 362. Of seven breast cancer patients (age 38–66) with a family history of breast cancer, one responded as normal with a frequency of 48 while the remaining six had a frequency ranging from 116 to 158 (Figure 1).

Cells from the one familial breast cancer patient showing a normal response to X-irradiation (chromatid break frequency <60, Figure 1) were exposed to UV-C (12 J) another DNA-damaging agent. Table III shows that cells from this patient have an ara-C effect of 2 compared with 44–80 for cells from six cancer-free donors. This result indicates an abnormally low incision activity for removal of UV-induced DNA damage.

### Discussion

Frequencies of chromatid breaks in blood lymphocytes from 9 of 11 individuals with a family history of breast cancer, six with breast cancer, one with preinvasive breast lesions and two with no cancer, was 2–3 fold higher after $G_2$ phase X-irradiation than that in cells from donors with no family history of breast cancer. Of the two individuals with a family history and a low chromatid break frequency (<60 after $G_2$ X-irradiation), only one had cancer. Cells from this individual after UV-C exposure, like those from XP-A, showed a minimal increase in chromatid breaks with ara-C treatment (Table III). XP-A and C cells are known to be defective in incision and removal of cyclobutane pyrimidine dimers in the overall genome (Evans *et al*., 1993). XP-A cells are also defective in removal of dimers from an active gene, whereas XP-C cells remove dimers normally from the transcribed strand of an active gene, but the non-transcribed strand is not repaired significantly in these cells (Evans *et al*., 1993). Thus, the responses of XP cells to X-irradiation in the chromosomal assay correlate to some extent with their responses to UV-induced damage. The ara-C effects of XP-C and XP-A cells, expressed as 17% and 2.4% of normal respectively, correlate extremely well with levels of repair of UV-induced damage of 10–20% and 1% respectively, (Table I), as measured by unscheduled DNA synthesis (UDS) (Robbins *et al*., 1974; Kraemer *et al*., 1975 and Petinga *et al*., 1977). Furthermore, the ara-C effect in CHO cells with or without human DNA repair genes correlates with the known incision activity of the cells. XP cells, usually resistant to

**Figure 1** Chromatid breaks in PHA-stimulated blood lymphocytes after X-ray-induced DNA damage during $G_2$. *Reported previously in Knight *et al*. (1993).

**Table II** Effect of ara-C (50 μM) on chromatid breaks in UV-C-irradiated (10 J) CHO cells with and without transfected human repair genes

| | Chromatid breaks per 100 metaphase cells | | | |
|---|---|---|---|---|
| *Cell line* | *Transfected gene* | *No ara-C* | *Ara-C*[a] | *Ara-C effect* |
| 27-1 | – | 30 | 26 | −4 |
| | ERCC-3 (XPB) | 8 | 86 | 78 |
| UV-61 | – | 14 | 16 | 2 |
| | ERCC-6 (CSB) | 10 | 108 | 98 |

[a]Ara-C (50 μM) added with colcemid 0.5–1.5 h after irradiation.

**Table III** Effect of ara-C on frequency of chromatid breaks in blood lymphocytes from normal donors and breast cancer patient after UV-C exposure

| | Chromatid breaks per 100 metaphase cells | | |
|---|---|---|---|
| *Donor* | *No Ara-C* | *Ara-C*[a] | *Ara-C Effect* |
| Normal | | | |
| 13651 | 6 | 86 | 80 |
| 13662 | 3 (2,4) | 68 (66,70) | 65 |
| 13681 | 6 | 50 | 44 |
| 13792 | 2 | 50 | 48 |
| 13884 | 2 | 54 | 52 |
| 13926 | 2 | 62 | 60 |
| Cancer patient | | | |
| 13 927 | 10 | 12 | 2 |

[a]Ara-C (50 μM) added at 10 min after UV and colcemid at 30 min after irradiation for 2 h.

ionising radiation, are thought to repair X-ray lesions normally. However, Satoh et al. (1993) reported a DNA excision-repair defect in XP (complementation groups A, B, C) that prevents removal of at least one type of oxygen free radical-induced base lesion. These lesions are repaired by the nucleotide excision repair (NER) process. NER requires enzymatic incision of the damaged DNA strand, removal of the damaged and neighboring nucleotides, repair replication by a polymerase to fill the space and ligation of the strand (Preston, 1980; Squires and Johnson, 1988; Hoeijmakers and Bootsma, 1994). Inhibition of the polymerase step by ara-C results in an accumulation of DNA strand breaks which, if unrepaired, are processed into chromatid breaks manifest at the subsequent metaphase (Natarajan et al., 1980; Preston, 1980). In the absence of ara-C, the high frequency of chromatid breaks in XP-A and C cells after X-irradiation (Table I) indicates a deficiency in repair of DNA strand breaks, either those produced directly by the X-irradiation or, more likely, because of the repair kinetics, those arising later during NER. The deficiency could be in ligation or in any one of the enzymatic steps following incision that leads to ligation. This chromosomal response was not seen, with few exceptions, in cells from normal healthy control donors.

Thus, it appears that cells from 10 of 11 individuals with a family history of breast cancer have a deficiency in the processing of X-ray- or UV-induced DNA damage, manifest as an abnormally high frequency of chromatid breaks or a reduced capacity for DNA incision, an early step in NER. A similar deficiency in the repair of X-ray-induced damage is reported in skin fibroblasts or peripheral blood lymphocytes from 56 obligate ataxia telangiectasia heterozygotes (Parshad et al., 1985; Sanford et al., 1990; Scott et al., 1994), who are at a 4-fold (range 2- to 7-fold) increase in relative risk of breast cancer (Easton, 1994). Furthermore, in a family with Li–Fraumeni syndrome, members with diverse cancers including those of breast, brain and other soft tissue showed a similar response to X-ray-induced DNA damage (Parshad et al., 1993a). One affected individual in this family was clinically normal at the time of biopsy to initiate the cell line studied, but developed two primary breast cancers later. Members of this particular family also carry a p53 germline mutation (Srivastava et al., 1990). Mutated p53 has been associated with breast and other cancers (Buller et al., 1993; Eyfjörd et al., 1995), moreover, it has recently been implicated in the repair of DNA damage (Smith et al., 1995; Wang et al., 1995) and genomic instability in primary human breast carcinomas (Eyfjörd et al., 1995). Furthermore, we showed previously that epithelial cells derived from human skin or normal human mammary tissue acquired a deficiency in repair of X-ray-induced DNA damage before they could be transformed in culture by chemicals or viruses to malignant cells, as evidenced by their growth as carcinomas in nude mice. These results seem to indicate that deficient DNA repair is a prerequisite for carcinogenesis (Gantt et al., 1987; Sanford et al., 1992; Parshad et al., 1994). Thus, deficient DNA repair, manifest as an abnormally high frequency of chromatid breaks or low activity for incision of UV-induced DNA damage, appears to be a predisposing factor in familial breast cancer. As some women with sporadic breast cancer also have such a deficiency, as shown here and earlier (Scott et al., 1994), deficient DNA repair may be a predisposing factor in the development of certain sporadic breast cancers.

**Acknowledgements**
The authors thank A M Denicoff, M Noone and M Gossard of the Division of Cancer Treatment for procuring the blood samples from patients and control donors.

### References

BULLER RE, ANDERSON B, CONNOR JP AND ROBINSON R. (1993). Familial ovarian cancer. *Gynecol. Oncol.*, **51**, 160–166.

COLLINS AR. (1993). Mutant rodent cell lines sensitive to ultraviolet light, ionizing radiation and cross-linking agents: a comprehensive survey of genetic and biochemical characteristics. *Mutat. Res.*, **293**, 99–118.

DEBAUCHE DM, PAI GS AND STANLEY WS. (1990). Enhanced $G_2$ chromatid radiosensitivity in dyskeratosis congenita fibroblasts. *Am. J. Human Genet.*, **46**, 350–357.

EASTON DF. (1994). Cancer risks in A–T heterozygotes. *Int. J. Radiat. Biol.*, **64** (suppl.), **S**, 177–182.

EVANS MK, ROBBINS JH, GANGES MB, TARONE RE, NAIRN RS AND BOHR VA. (1993). Gene-specific DNA repair in xeroderma pigmentosum complementation groups A, C, D and F. *J. Biol. Chem.*, **268**, 4839–4847.

EYFJÖRD JE, THORLACIUS S, STEINDARSDOTTIR M, VALGARDSDOTTIR R, ÖGMUNDSDOTTIR HM AND ANAMTHAWAT-JONSSON K. (1995). p53 abnormalities and genomic instability in primary human breast carcinomas. *Cancer Res.*, **55**, 646–651.

FRIEDBERG EC, WALKER GC AND SIEDE W. (1995). *DNA Repair and Mutagenesis.* ASM Press: Washington, DC.

GANTT R, SANFORD KK, PARSHAD R, PRICE FM, PETERSON WD AND RHIM JS. (1987). Enhanced $G_2$ chromatid radiosensitivity, an early stage in the neoplastic transformation of human epidermal keratinocytes in culture. *Cancer Res.*, **47**, 1390–1397.

HOEIJMAKERS JHJ AND BOOTSMA D. (1994). Incision for excision. *Nature*, **371**, 654–655.

KNIGHT RB, PARSHAD R, PRICE FM, TARONE RE AND SANFORD KK. (1993). X-ray-induced chromatid damage in relation to DNA repair and cancer incidence in family members. *Int. J. Cancer*, **54**, 589–593.

KRAEMER KH, COON HG, PETINGA RA, BARRETT SF, RAHE A AND ROBBINS JH. (1975). Genetic heterogeneity in xeroderma pigmentosum: complementation groups and their relationship to DNA repair rates. *Proc. Natl Acad. Sci., USA*, **72**, 59–63.

MOZDARANI H AND BRYANT PE. (1989). Kinetics of chromatid aberrations in $G_2$ ataxia-telangectasia cells exposed to x-rays and ara-A. *Int. J. Radiat. Biol.*, **55**, 71–84.

NATARAJAN AT, OBE G, VAN ZEELAND AA, PALITTI F, MEIJERS M AND VERDEGAAL-IMMERZEEL EA. (1980). Molecular mechanisms involved in the production of chromosomal aberration. II. Utilization of neurospora endonuclease for the study of aberration production by x-ray in $G_1$ and $G_2$ studies of cell cycle. *Mutat. Res.*, **69**, 293–305.

PANDITA TK AND HITTELMAN WN. (1995). Evidence of a chromatin basis for increased mutagen sensitivity associated with multiple primary malignancies of the head and neck. *Int. J. Cancer*, **61**, 738–743.

PARSHAD R, SANFORD KK AND JONES GM. (1983). Chromatid damage after $G_2$ phase x-irradiation of cells from cancer-prone individuals implicates deficiency in DNA repair. *Proc. Natl Acad. Sci. USA*, **80**, 5612–5616.

PARSHAD R, SANFORD KK, JONES GM AND TARONE RE. (1985). $G_2$ chromosomal radiosensitivity of ataxia-telangiectasia heterozygotes. *Cancer Genet. Cytoget.*, **14**, 163–168.

PARSHAD R, PRICE FM, PIROLLO KF, CHANG EH AND SANFORD KK. (1993*a*). Cytogenetic response to $G_2$-phase x-irradiation in relation to DNA repair and radiosensitivity in a cancer-prone family with Li–Fraumeni syndrome. *Radiat. Res.*, **136**, 236–240.

PARSHAD R, TARONE RE, PRICE FM AND SANFORD KK. (1993*b*). Cytogenetic evidence for differences in DNA incision activity in xeroderma pigmentosum groups A, C, and D cells after x-irradiation during $G_2$ phase. *Mutat. Res.*, **294**, 149–155.

PARSHAD R, SANFORD KK, PRICE FM, RHIM JS, TARONE RE, FUSENIG NE AND BOUKAMP P. (1994). Association of deficient DNA repair during $G_2$ phase with progression from benign to malignant state in a line of human skin keratinocytes transfected with ras oncogene. *Carcinogenesis*, **15**, 33–37.

PETINGA RA, ANDREWS AD, TARONE RE AND ROBBINS JH. (1977). Typical xeroderma pigmentosum complementation group A fibroblasts have detectable ultraviolet light-induced unscheduled DNA synthesis. *Biochim. Biophys. Acta*, **479,** 400–419.

PRESTON GA, PAYNE HS AND PRESTON RJ. (1992). Isolation and characterization of a 1-$\beta$-D-arabinofuranosylcytosine-resistant Chinese hamster ovary cell mutant that is also x-ray sensitive and is non-complementary with ataxia telangiectasia cells. *Cancer Res.*, **52,** 319–329.

PRESTON RJ. (1980). The effect of cytosine arabinoside on the frequency of x-ray-induced chromosome aberrations in normal human leukocytes. *Mutat. Res.*, **69,** 71–79.

ROBBINS JH, KRAEMER KH, LUTZNER MA, FESTOFF BW AND COON HG. (1974). An inherited disease with sun sensitivity, multiple cutaneous neoplasms and abnormal DNA repair. *Ann. Intern. Med.*, **80,** 221–248.

SANFORD KK, PARSHAD R, GREENE MH, TARONE RE, TUCKER MA AND JONES GM. (1987). Hypersensitivity to $G_2$ chromatid radiation damage in familial dysplastic nevus syndrome. *Lancet*, **2,** 1111–1116.

SANFORD KK, PARSHAD R, GANTT R, TARONE RE, JONES GM AND PRICE FM. (1989). Factors affecting and significance of $G_2$ chromatin radiosensitivity in predisposition to cancer. *Int. J. Radiat. Biol.*, **55,** 963–981.

SANFORD KK, PARSHAD R, PRICE FM, JONES GM, TARONE RE, EIERMAN L, HALE P AND WALDMAN TA. (1990). Enhanced chromatin damage in blood lymphocytes after $G_2$ phase x-irradiation, a marker of the ataxia-telangiectasia gene. *J. Natl Cancer Inst.*, **82,** 1050–1054.

SANFORD KK, PARSHAD R, PRICE FM, RHIM JS, STAMPFER M AND PARSHAD R. (1992). Role of DNA repair in malignant neoplastic transformation of human mammary epithelial cells in culture. *Carcinogenesis*, **13,** 1137–1141.

SANFORD KK, PARSHAD R, PRICE FM, TARONE RE AND SCHAPIRO MB. (1993). X-ray-induced chromatid damage in cells from Down syndrome and Alzheimer disease patients in relation to DNA repair and cancer proneness. *Cancer Genet. Cytogenet.* **70,** 25–30.

SATOH MS, JONES CJ, WOOD RD AND LINDAHL RT. (1993). DNA excision-repair defect in xeroderma pigmentosum prevents removal of a class of oxygen free radical-induced base lesions. *Proc. Natl Acad. Sci. USA*, **90,** 6335–6339.

SCOTT D, SPREADBOROUGH A, LEVINE E AND ROBERTS SA. (1994). Genetic predisposition in breast cancer. *Lancet*, **344,** 1444.

SMITH, ML, CHEN I-T, ZHAN Q, O'CONNOR PM AND FORNACE AJ, JR. (1995). Involvement of the p53 tumor suppressor in repair of UV-type DNA damage. *Oncogene*, **10,** 1053–1059.

SQUIRES S AND JOHNSON RT. (1988). Kinetic analysis of UV-induced incision discriminates between fibroblasts from different xeroderma pigmentosum complementation groups, XPA heterozygotes and normal individuals. *Mutat. Res.*, **193,** 181–192.

SRIVASTAVA S, ZOU Z, PIROLLO K, BLATTNER W AND CHANG EH. (1990). Germ-line transmission of a mutated p53 gene in a cancer-prone family with Li–Fraumeni syndrome. *Nature*, **348,** 747–749.

TAKAI S, PRICE FM, SANFORD KK, TARONE RE AND PARSHAD R. (1990). Persistence of chromatid damage after $G_2$ phase x-irradiation in lymphoblastoid cells from Gardner's syndrome. *Carcinogenesis*, **11,** 1425–1428.

TROELSTRA C, VAN GOOL A, DE WIT J, VERMEULEN W, BOOTSMA D AND HOEIJMAKERS HJ. (1992). ERCC6, a member of a subfamily of putative helicases, is involved in Cockayne's syndrome and preferential repair of active genes. *Cell*, **71,** 939–953.

TROELSTRA C, HESEN W, BOOTSMA D AND HOEIJMAKERS JHJ. (1993). Structure and expression of the excision repair gene ERCC6, involved in the human disorder Cockayne's syndrome group *B. Nucleic Acids Res.*, **21,** 419–426.

VERMUELEN W, SCOTT RJ, RODGERS S, MÜLLER HJ, COLE J, ARLETT CF, KLEIJER WJ, BOOTSMA D, HOEIJMAKERS JHJ AND WEEDA G. (1994). Clinical heterogeneity within xeroderma pigmentosum associated with mutations in the DNA repair and transcription gene ERCC3. *Am. J. Human. Genet.*, **54,** 191–200.

WANG XW, YEH H, SCHAEFFER L, ROY R, MONCOLLIN V, EGLY J-M, WANG Z, FRIEDBERG EC, EVANS MK, TAFFE BG, BOHR VA, WEEDA G, HOEIJMAKER JHJ, FORRESTER K AND HARRIS CC. (1995). p53 modulation of TFIIH-associated nucleotide excision repair activity. *Nature Genet.*, **10,** 188–195.

WEEDA D, VAN HAM RCA, VERMEULEN W, BOOTSMA D, VAN DER EB AJ AND HOEIJMAKERS HJ. (1990). A presumed DNA helicase encoded by ERCC-3 is involved in the human repair disorders xeroderma pigmentosum and Cockayne's syndrome. *Cell*, **62,** 777–791.